# Iron deficiency and biomarkers of inflammation: a 3-year prospective analysis of the DO-HEALTH trial

**DOI:** 10.1007/s40520-021-01955-3

**Published:** 2021-09-17

**Authors:** Maud Wieczorek, Franziska Schwarz, Angélique Sadlon, Lauren A. Abderhalden, Caroline de Godoi Rezende Costa Molino, Donat R. Spahn, Dominik J. Schaer, E. John Orav, Andreas Egli, Heike A. Bischoff-Ferrari, DO-HEALTH Research group

**Affiliations:** 1 Department of Aging Medicine and Aging Research, University Hospital Zurich and University of Zurich, Raemistrasse 101, 8091 Zurich, Switzerland; 2 Centre on Aging and Mobility, University Hospital Zurich and City Hospital Zurich, Waid, Zurich, Switzerland; 3 Ageing Epidemiology (AGE) Research Unit, Imperial College London, London, UK; 4 Institute of Anesthesiology, University Hospital Zurich and University of Zurich, Zurich, Switzerland; 5 Division of Internal Medicine, University Hospital Zurich and University of Zurich, Zurich, Switzerland; 6 Department of Biostatistics, Harvard School of Public Health, Boston, USA; 7 University Clinic for Aging Medicine, City Hospital Zurich, Waid, Zurich, Switzerland

**Keywords:** Iron deficiency, Anemia, Hs-CRP, IL-6, Inflammaging, DO-HEALTH

## Abstract

**Background:**

The longitudinal association between iron deficiency and inflammatory biomarkers levels has not been fully explored among relatively healthy older adults.

**Aims:**

To assess whether iron deficiency at baseline and at any yearly follow-up time point, with or without anemia, was associated with changes from baseline in high-sensitivity C-reactive protein (hs-CRP) and interleukin-6 (IL-6) levels over 3 years.

**Methods:**

This is a post-hoc observational analysis of DO-HEALTH, a double-blind, randomized controlled trial including 2157 European community-dwelling adults age 70+. The outcomes were changes from baseline in hs-CRP and IL-6 levels, measured at 12, 24, and 36 months of follow-up. Iron deficiency was defined by soluble transferrin receptor levels > 28.1 nmol/L and baseline anemia by hemoglobin levels < 130 g/L for men and < 120 g/L for women.

**Results:**

In total, 2141 participants were included in the analyses (mean age: 74.9 years, 61.5% of women, 26.8% with iron deficiency). Baseline iron deficiency was associated with greater increase in IL-6 levels (mean difference in change: 0.52 ng/L, 95%CI 0.03–1.00, *P* = .04) over 3 years. Iron deficiency at any yearly time point was associated with higher increases in hs-CRP (mean difference in change: 1.62 mg/L, 95%CI 0.98–2.26, *P* < .001) and IL-6 levels (mean difference in change: 1.33 ng/L, 95%CI 0.87–1.79, *P* < .001) over 3 years. No significant interaction between iron deficiency and anemia was found, suggesting that the results are independent of the anemic status.

**Conclusions:**

These findings suggest that iron deficiency may play a role in low-grade chronic inflammation among relatively healthy older adults.

**Supplementary Information:**

The online version contains supplementary material available at 10.1007/s40520-021-01955-3.

## Introduction

Iron deficiency (ID) is the most frequent nutritional disorder worldwide affecting populations of all ages, sexes, and ethnic descent. [1–3] Iron is an essential micronutrient and its homeostasis plays an important role in several physiological human processes, including cellular activities, erythropoiesis, and innate immune function. [4] Inflammatory stimuli induce macrophage production of interleukin-6 (IL-6), which stimulates hepatocytes to produce acute-phase proteins including C-reactive protein (CRP) [5, 6] and hepcidin. [7] This inflammation state may lead to extended hypoferremia and cause anemia. [8]

While there is good scientific evidence that inflammation may predict ID, evidence on the influence of ID on pre-existing low-grade inflammation remains limited. With aging, loss of homeostasis alters the production of cytokines, leading to a low-grade chronic systemic pro-inflammatory state characterized by permanently elevated levels of IL-6 and CRP. This state, also called “inflammaging” [9], is considered to be a promotor of accelerated aging [10], age-related syndromes such as frailty [11–14], and mortality [15–18] in older adults. Thus, capturing iron status among older adults may be relevant to the promotion of healthy aging and the prevention of age-related chronic diseases.

Recently, high prevalence of ID was reported in a population of community-dwelling older adults. [19] To our knowledge, so far, no longitudinal prospective study has investigated the association between ID and low-grade chronic inflammation in relatively healthy older individuals. In a recent cross-sectional study, iron-deficient women with mean age 58 years showed increased levels of CRP and IL-6 compared to healthy controls. [20] However, the use of a different ID definition prevents the accurate comparison of results between studies. [21, 22]

Therefore, the aim of the present study was to assess whether the presence of ID at baseline and at yearly follow-up visits, with or without anemia, was associated with changes from baseline in high-sensitivity CRP (hs-CRP) and IL-6 levels over 3 years in a large European cohort of relatively healthy community-dwelling older adults.

## Methods

### Study design and participants

The study is a post-hoc observational analysis of the DO-HEALTH clinical trial with a prospective, longitudinal approach. DO-HEALTH is a multi-center, double-blind, randomized controlled clinical trial designed to support healthy aging in European older adults (NCT01745263). The trial examined the individual and combined effects of omega-3 fatty acids, vitamin D, and a simple home exercise program over 3 years of follow-up. A total of 2157 community-dwelling healthy and pre-frail seniors aged 70 years and older were recruited from 7 centers in 5 European countries: Zurich, Basel, Geneva (Switzerland), Berlin (Germany), Innsbruck (Austria), Toulouse (France), and Coimbra (Portugal). Inclusion criteria were absence of major health events in the 5 years prior to enrollment, sufficient mobility, and good cognitive status. Further details are provided elsewhere. [23]

### Primary outcomes: biomarkers of inflammation

For the present study, the 3-year-follow-up data were used. Fasting blood samples, collected in the morning were taken at baseline, 12, 24, and 36 months of follow-up. Hs-CRP levels were measured with the C-Reactive Protein Gen. 3 test on a cobas e 701 analyser (Roche) using an Immunoturbidimetric assay. IL-6 levels were measured using the Elecsys IL-6 assay on cobas e 801 analyser (Roche) with ElektroChemiLumineszenz-ImmunoAssay “ECLIA” technology. Higher levels of hs-CRP and IL-6 indicate greater inflammation.

### Exposures: ID and anemia

We used soluble transferrin receptor levels (sTfR) levels as a clinical marker of ID at baseline and over the follow-up, since this parameter is not influenced by inflammation. [24] The threshold of more than 28.1 nmol/L was used to define ID as it was validated in older adults. [25–27] sTfR levels were measured with Tina-quant Transferrin ver.2 Test on a cobas c 502 analyser (Roche) using an Immunoturbidimetric assay. Considering the lack of consensus in the definitions of ID [7], we performed sensitivity analyses using serum ferritin levels and the sTfR-ferritin index (sTfR / log Ferritin) as alternate definitions of ID. Ferritin concentrations were measured with Elecsys Ferritin Test on a cobas e 801 analyser (Roche) using ElektroChemiLumineszenz-ImmunoAssay "ECLIA" technology. ID was then defined using the established cutoffs of ferritin less than 45 μg/L [28, 29] and 30 μg/L for older adults [30, 31]; and of sTfR-ferritin index over 1.5 ng/mL. [26, 32]

Hemoglobin was measured in the whole blood at baseline. We used hemoglobin levels less than 130 g/L for men and less than 120 g/L for women to define anemia, according to the World Health Organization guidelines. [33]

### Baseline covariates

Participants’ characteristics such as age, sex, tobacco consumption, and body mass index (BMI) were collected at baseline. Alcohol consumption (g/day) was derived from the Food Frequency Questionnaire. [34] Comorbidities were assessed with the self-administered comorbidity questionnaire. [35] Frailty status was determined according to Fried criteria. [36] Polypharmacy was defined as the use of 5 or more medications. Frequency of physical activity (0, 1–2, ≥ 3 times per week) was measured using the Nurses’ Health Study questionnaire. [37]

### Statistical analysis

Baseline demographic and clinical characteristics, including baseline hs-CRP and IL-6 levels, were compared between ID and non-ID participants using Chi-square and *t* tests. Linear regression models based on generalized estimating equations (GEE) for repeated measurements were used to compare changes from baseline in hs-CRP and IL-6 levels in participants who were ID versus non-ID at baseline. Each participant had up to 3 outcome measures (hs-CRP or IL-6 changes from baseline at 1, 2, and 3 years) and the primary predictors were baseline ID status, follow-up year, and the interactions between these two predictors. Models were adjusted for treatment allocation, study site and baseline covariates: age [38], sex [39], number of comorbidities [22], alcohol consumption [40], tobacco consumption [41], polypharmacy [22], frailty status [11, 13], frequency of physical activity [5], and the baseline level of the outcome. Time-varying BMI [5] and the incidence rate of infections [42] (number of infections per year/person-days per year) in the year preceding the measurement of the inflammatory biomarkers were also used as adjustment variables. These models allowed us to estimate, at each yearly follow-up, whether the change from baseline in hs-CRP or IL-6 level was different between participants who were ID compared to non-ID. Additionally, we estimated the effect of baseline ID status on hs-CRP and IL-6 changes across all follow-up times simultaneously. Finally, to explore the effect of the presence of ID at each follow-up (rather than at baseline), we included a time-varying variable for iron status in separate, analogous GEE models controlling for baseline iron status. Mean differences in changes (MD) with 95% confidence intervals (CI) are presented.

In exploratory analyses, for each outcome, interactions between ID and age (70–74 years, 75 years and older), sex, and anemic status were tested. A sensitivity analysis was performed to investigate the association between recurrent iron deficiency (defined as sTfR levels > 28.1 nmol/L at 2 or more consecutive time points vs 1 time point—including baseline) and biomarkers levels. Furthermore, since liver and kidneys perform a major role in iron homeostasis [43–46], we excluded participants who reported baseline liver disease (*n* = 37), kidney disease (*n* = 54), and participants with incident invasive cancer over the follow-up (*n* = 74) in a second sensitivity analysis.

Statistical analyses involved using SAS version 9.4 (SAS Institute, Cary, NC). Two-sided *p* values < 0.05 were considered statistically significant.

## Results

### Baseline characteristics of the study population

Of the 2157 trial participants, 16 (0.7%) had missing baseline sTfR levels. In total, 2141 participants were included in the analyses. At baseline, 573 (26.8%) of them suffered from ID, and over the 3-year follow-up, a total of 262 new cases of ID were identified among non-ID participants at baseline. Baseline characteristics of subjects are presented in Table 1. Overall, the mean age was 74.9 (4.5) years, including 1317 (61.5%) women. The mean levels of hs-CRP and IL-6 were 2.9 mg/L (5.6) and 3.8 ng/L (6.9), respectively. At baseline, ID participants were more likely to be older (*P* < 0.001), less physically active (*P* = 0.009), at least pre-frail (*P* = 0.007), subject to polypharmacy (*P* < 0.001), and anemic (*P* < 0.001). In addition, they were more likely to have fewer years of education (*P* < 0.001), a higher BMI (*P* < 0.001), lower alcohol consumption (*P* = 0.008), and more comorbidities (*P* < 0.001), compared to the non-ID participants.

**Table. 1:** Baseline characteristics of the study population

	Baseline iron deficiency	No baseline iron deficiency	*p* value ^a^	Overall
	sTfR > 28.1 nmol/L	sTfR ≤ 28.1 nmol/L		
	*n* = 573 (26.8)	*n* = 1568 (73.2)		*n* = 2141
Sex, *N* (%)			0.29	
Women	363 (63.4)	954 (60.8)		1317 (61.5)
Men	210 (36.7)	614 (39.2)		824 (38.5)
Age, mean (SD), years	75.6 (4.7)	74.7 (4.3)	< 0.001	74.9 (4.5)
Education, years	12.1 (4.4)	12.9 (4.2)	< 0.001	12.7 (4.3)
BMI, mean (SD), kg/m^2^	27.1 (4.5)	26.0 (4.2)	< 0.001	26.3 (4.3)
Alcohol, mean (SD), g/day	7.7 (11.1)	9.3 (11.9)	0.008	8.9 (11.7)
Current smokers, *N* (%)	14 (2.4)	111 (7.1)	< 0.001	125 (5.8)
Live alone, *N* (%)	242 (42.2)	655 (41.8)	0.85	897 (41.9)
Physical activity, *N* (%)			0.009	
None	123 (21.5)	249 (15.9)		372 (17.4)
1–2 times per week	170 (29.7)	474 (30.3)		644 (30.1)
≥ 3 times per week	280 (48.9)	843 (53.8)		1123 (52.5)
Frailty status ^b^, *N* (%)			0.007	
Robust	272 (48.4)	852 (55.1)		1124 (53.3)
At least pre-frail	290 (51.6)	695 (44.9)		985 (46.7)
Polypharmacy ^c^, *N* (%)	191 (33.3)	384 (24.5)	< 0.001	575 (26.9)
Iron supplementation, *N* (%)	37 (6.5)	83 (5.3)	0.30	120 (5.6)
Number of comorbidities ^d^, mean (SD)	2 (1.5)	1.6 (1.4)	< 0.001	1.7 (1.4)
Anemia ^e^, *N* (%)	63 (11.0)	77 (4.9)	< 0.001	140 (6.5)
hs-CRP, mean (SD), mg/L	4.0 (8.4)	2.5 (4.2)	< 0.001	2.9 (5.6)
IL-6, mean (SD), ng/L	4.7 (9.3)	3.5 (5.9)	0.003	3.8 (6.9)
Countries, *N* (%)			0.02	
Austria	55 (9.6)	143 (9.1)		198 (9.3)
France	73 (12.7)	226 (14.1)		299 (14.0)
Germany	92 (16.1)	254 (16.2)		346 (16.2)
Portugal	101 (17.6)	192 (12.2)		293 (13.7)
Switzerland	252 (44.0)	753 (48.0)		1005 (46.9)

*BMI* Body Mass Index, *sTfR* soluble Transferrin Receptor, *hs-CRP* high-sensitivity C-Reactive Protein, *IL-6* Interleukin-6

^a^ Differences between iron-deficient and non-iron-deficient participants at baseline were assessed by an independent *t* test for continuous variables and Chi-square test for categorical variables.

^b^Frailty status was defined using the Fried Physical Frailty Phenotype which evaluates 5 criteria: fatigue (self-reported), unintentional weight loss (self-reported loss more than 5% of total body weight), reduced physical activity (self-reported), slowness (impaired walking speed) and weakness (low grip strength). Participants are classified as at least pre-frail when one or more of the criteria are presented, and otherwise classified as robust.

^c^ Polypharmacy was defined as the concomitant use of 5 or more medications.

^d^ Self-reported number of comorbidities was assessed by the Sangha questionnaire, range 0–13.

^e^ Anemia was defined as hemoglobin < 130 g/L for men and < 120 g/L for women.

### Association between baseline ID and inflammatory biomarkers

**Fig 1:**
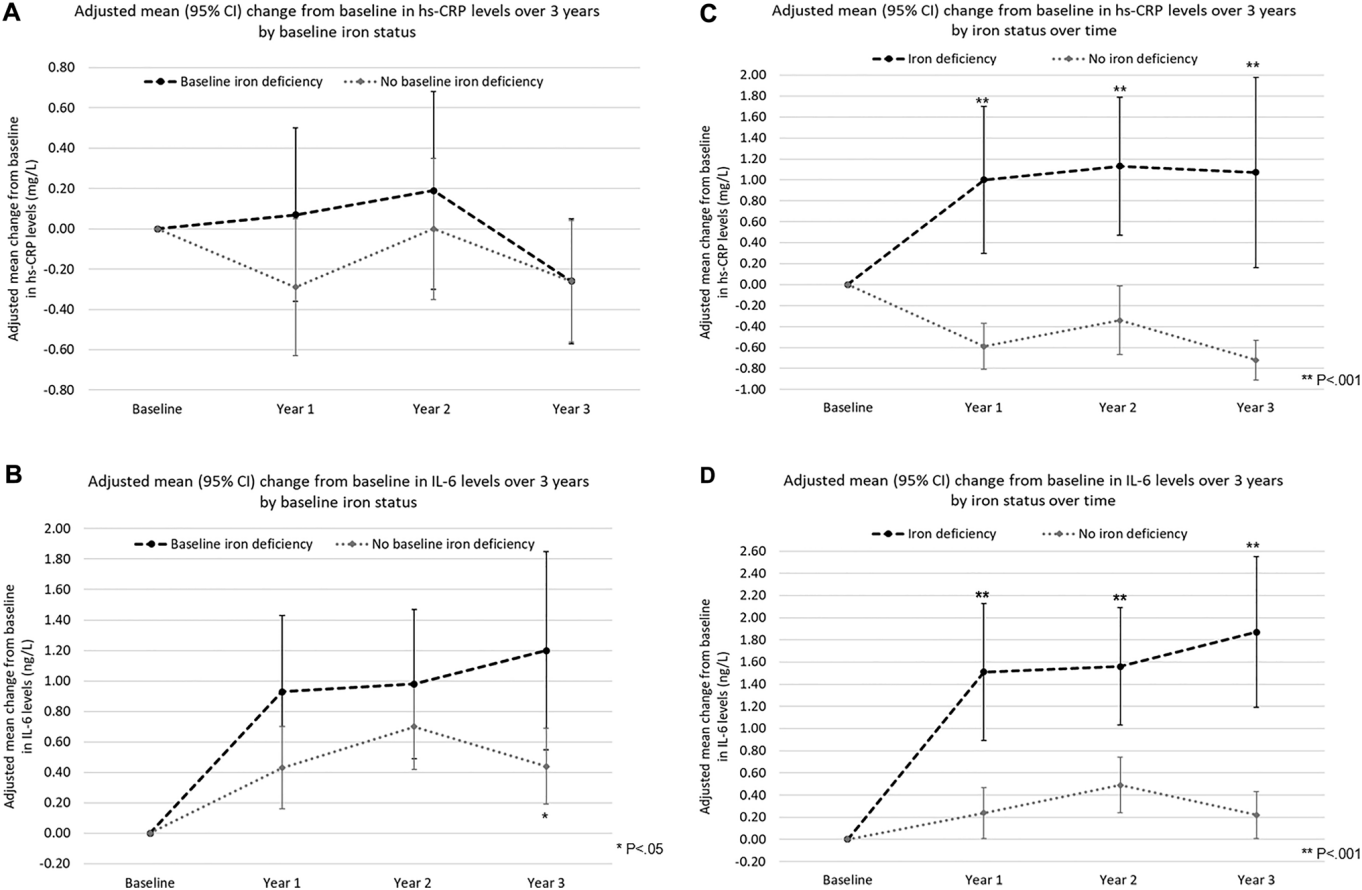
Changes in inflammatory biomarkers levels over time. The adjusted mean change from baseline over three years is shown for (**a**) hs-CRP levels by baseline iron status, **b** IL-6 levels by baseline iron status, **c** hs-CRP levels by iron status at each yearly time point over the follow-up, and **d** IL-6 levels by iron status at each yearly time point over the follow-up

Overall, a significant difference in baseline hs-CRP levels was observed according to the iron status, with higher levels in ID subjects (unadjusted MD: 1.49 mg/L, 95% CI: 0.78–2.21, *P* < 0.001) (Table 2). However, there was no statistically significant difference in hs-CRP levels over time between participants with ID and non-ID at baseline (Fig. 1a).

**Table. 2:** Changes from baseline in hs-CRP and IL-6 levels by baseline iron status

	Baseline iron deficiency sTfR levels > 28.1 nmol/L	No baseline iron deficiency sTfR levels ≤ 28.1 nmol/L	Mean difference in change from baseline (95% CI)	P value for mean difference in change from baseline between groups ^a^
	*n* = 573	*n* = 1568		
hs-CRP (mg/L)				
Unadjusted at baseline, mean (SD)	3.99 (0.35)	2.49 (0.10)	**1.49 (0.78 to 2.21)**	** < 0.001**
Adjusted change at Year 1 (95% CI)	0.07 (** − **0.37 to 0.50)	** − 0.29 ( − 0.54 to − 0.05)**	0.36 (0.15 to 0.87)	0.16
Adjusted change at Year 2 (95% CI)	0.19 (** − **0.29 to 0.68)	0.00 (-0.35 to 0.35)	0.19 (** − **0.40 to 0.79)	0.52
Adjusted change at Year 3 (95% CI)	** − **0.26 (** − **0.57 to 0.05)	** − **0.26 (** − **0.56 to 0.04)	** − **0.01 (** − **0.44 to 0.43)	0.98
Adjusted change across all time points (95% CI)	** − **0.00 (** − **0.28 to 0.28)	** − **0.18 (** − **0.38 to 0.01)	0.18 (** − **0.16 to 0.52)	0.29
IL-6 (ng/L)				
Unadjusted at baseline, mean (SD)	4.72 (0.39)	3.46 (0.15)	**1.26 (0.45 to 2.07)**	**0.003**
Adjusted change at Year 1 (95% CI)	**0.93 (0.43 to 1.43)**	**0.43 (0.15 to 0.70)**	0.51 (** − **0.09 to 1.10)	0.09
Adjusted change at Year 2 (95% CI)	**0.98 (0.50 to 1.47)**	**0.70 (0.41 to 0.98)**	0.29 (** − **0.31 to 0.89)	0.35
Adjusted change at Year 3 (95% CI)	**1.20 (0.55 to 1.85)**	**0.44 (0.20 to 0.69)**	**0.75 (0.03 to 1.48)**	0.04
Adjusted change across all time points (95% CI)	**1.04 (0.65 to 1.43)**	**0.52 (0.31 to 0.73)**	**0.52 (0.03 to 1.00)**	**0.04**

^a^ *p* values correspond to the mean differences in biomarkers levels or changes in biomarker levels between iron-deficient and non-iron-deficient groups. Baseline levels are compared using a *t* test. Yearly changes from baseline are compared by repeated measures linear regression with interaction terms between iron deficiency status and time. Overall differences across all time points are compared by repeated measures linear regression with a main effect for iron deficiency

Models are adjusted for treatment allocation, age, sex, center, body mass index over the follow-up, alcohol consumption, tobacco consumption, polypharmacy, number of comorbidities, frailty status (pre-frailty), frequency of physical activity, yearly incidence rate of infections, and baseline level of the outcome

*sTfR* soluble Transferrin Receptor

Values in bold indicate significant P values

Significantly higher baseline IL-6 levels were observed in ID participants compared to non-ID participants (unadjusted MD: 1.26 ng/L, 95% CI: 0.45–2.07, *P* = 0.003). Over time, we found a significant increase in IL-6 levels among both non-ID (0.52 ng/L) and ID participants (1.04 ng/L), with a significantly greater increase in the latter (MD: 0.52 ng/L, 95% CI: 0.03–1.00, *P* = 0.04) (Fig. 1b).

The interaction between ID and baseline anemic status was not statistically significant in hs-CRP (*P* = 0.23) and IL-6 models (*P* = 0.42), suggesting that results are independent of anemic status. A significant interaction between sex and baseline ID was found (*P* = 0.03). We observed a significant decrease in CRP levels between baseline and year 3 in male ID subjects, while there was no significant change in hs-CRP levels over time in both female subgroups (Online Resource 1). A significant interaction was also found between baseline ID and age groups (*P* = 0.01). In younger participants, IL-6 levels increased regardless the iron status, without any significant difference across all time points. In older subjects, ID was significantly associated with a greater increase in IL-6 levels over time (Online Resource 2).

### Association between yearly-assessed ID and inflammatory biomarkers

Participants with ID at any yearly follow-up time point had 1.59 mg/L higher concentration of hs-CRP at baseline (unadjusted, 95% CI 0.89–2.29, *P* < 0.001) (Table 3). Across all yearly follow-ups, there was a significant increase in hs-CRP levels among subjects with ID at a particular follow-up time point (1.07 mg/L), while there was a significant decrease in hs-CRP levels in participants who did not have ID at these time points ( − 0.55 mg/L). The presence of ID at a yearly follow-up time point was associated with a statistically significant greater increase in hs-CRP levels (MD: 1.62 mg/L, 95% CI 0.98–2.26, *P* < 0.001) (Fig. 1c).

**Table 3:** Changes from baseline in hs-CRP and IL-6 levels by yearly-assessed iron status

	Iron deficiency at any yearly follow-up time point sTfR levels > 28.1 nmol/L	No iron deficiency at any yearly follow-up time point sTfR levels ≤ 28.1 nmol/L	Mean difference in change from baseline (95% CI)	*P* value for mean difference in change from baseline between groups ^a^
hs-CRP (mg/L)				
Unadjusted at baseline, mean (SD)	4.05 (0.34)	2.47 (0.11)	**1.59 (0.89 to 2.29)**	** < 0.001**
Adjusted change at Year 1 (95% CI)	**1.00 (0.30 to 1.70)** [*n* = 481]	** − 0.59 (-0.81 to -0.37) **[*n* = 1419]	**1.59 (0.82 to 2.37)**	** < 0.001**
Adjusted change at Year 2 (95% CI)	**1.13 (0.47 to 1.79) **[*n* = 485]	** − 0.34 ( − 0.67 to − 0.01)** [*n* = 1346]	**1.47 (0.70 to 2.24)**	** < 0.001**
Adjusted change at Year 3 (95% CI)	**1.07 (0.17 to 1.98)** [*n* = 462]	** − 0.72 ( − 0.90 to − 0.53)** [*n* = 1367]	**1.80 (0.83 to 2.77)**	** < 0.001**
Adjusted change across all time points (95% CI)	**1.07 (0.50 to 1.63)**	** − 0.55 ( − 0.72 to − 0.38)**	**1.62 (0.98 to 2.26)**	** < 0.001**
IL-6 (ng/L)				
Unadjusted at baseline, mean (SD)	4.66 (0.33)	3.48 (0.15)	**1.19 (0.52 to 1.86)**	** < 0.001**
Adjusted change at Year 1 (95% CI)	**1.51 (0.88 to 2.13) **[*n* = 481]	**0.24 (0.01–0.47) **[*n* = 1419]	**1.27 (0.60 to 1.94)**	** < 0.001**
Adjusted change at Year 2 (95% CI)	**1.56 (1.04 to 2.09)** [*n* = 485]	**0.49 (0.24–0.74)** [*n* = 1346]	**1.07 (0.48 to 1.67)**	** < 0.001**
Adjusted change at Year 3 (95% CI)	**1.87 (1.18 to 2.55)** [*n* = 462]	**0.22 (0.01–0.43)** [*n* = 1367]	**1.65 (0.92 to 2.37)**	** < 0.001**
Adjusted change across all time points (95% CI)	**1.65 (1.23 to 2.06)**	**0.32 (0.14–0.49)**	**1.33 (0.87 to 1.79)**	** < 0.001**

^a^ *p* values correspond to the mean differences in biomarkers levels or changes in biomarker levels between iron-deficient and non-iron-deficient groups. Baseline levels are compared using a *t* test. Yearly changes from baseline are compared by repeated measures linear regression with interaction terms between iron deficiency status and time. Overall differences across all time points are compared by repeated measures linear regression with a main effect for iron deficiency

Models are adjusted for treatment allocation, age, sex, center, body mass index over the follow-up, alcohol consumption, tobacco consumption, polypharmacy, number of comorbidities, frailty status (pre-frailty), frequency of physical activity, yearly incidence rate of infections, baseline iron status and baseline level of the outcome

Numbers between squared brackets indicate the number of iron-deficient and non-iron-deficient participants at each yearly time point

*sTfR* soluble Transferrin Receptor

Values in bold indicate significant *P* values

At baseline, IL-6 levels were significantly higher in participants who had ID at any yearly follow-up time point (unadjusted MD: 1.19 ng/L, 95% CI 0.52–1.86, *P* < 0.001). Over time, we observed a significant increase in IL-6 levels among both participants with ID (1.65 ng/L) and without ID (0.32 ng/L). The presence of ID at any yearly time point over the follow-up was associated with a greater increase in IL-6 levels (MD: 1.33 mg/L, 95% CI 0.87–1.79, *P* < 0.001) (Fig. 1d).

We did not find any significant interaction between ID at any yearly follow-up time point and anemic status (*P* = 0.24 and *P* = 0.46 in hs-CRP and IL-6 models, respectively), suggesting that the results are independent of the presence of anemia. Results were similar for the interaction between ID at any yearly time point and sex (*P* = 0.25 in hs-CRP model and *P* = 0.72 in IL-6 model) or age groups (*P* = 0.59 in hs-CRP model and *P* = 0.32 in IL-6 model).

## Sensitivity analysis

### Association between baseline ID defined with ferritin levels and transferrin–ferritin index and inflammatory biomarkers

Over 3 years, no statistically significant difference over time in hs-CRP levels was found between ID and non-ID groups, when ID was defined by both < 45 μg/L and < 30 μg/L ferritin levels (Online Resource 3). IL-6 levels significantly increased in all subjects, without any significant difference between the 2 groups over time, regardless of the ferritin cut-off used.

When defining ID with transferrin–ferritin index, ID was associated with a greater increase in hs-CRP and IL-6 concentrations, across all time points (Online Resource 3).

### Association between ID defined with ferritin levels and transferrin–ferritin index over the follow-up and inflammatory biomarkers

Regardless of the cut-off applied for ferritin levels, the presence of ID at any yearly time point was associated with a decrease in hs-CRP levels across all time points (Online Resource 4). There was no significant difference in IL-6 levels between participants with ID at a particular time over the follow-up and those without. When defined by transferrin–ferritin index > 1.5, the presence of ID at any yearly time point over the follow-up was associated with a greater increase in IL-6 across all time points (Online Resource 4).

### Association between recurrent ID and inflammatory biomarkers

Across all time points, the presence of recurrent iron deficiency was associated with statistically significant greater increases in hs-CRP and IL-6 levels (Online Resource 5).

### Exclusion of participants with baseline liver, kidney disease, and incident invasive cancers

When excluding participants with baseline liver, kidney disease, and incident invasive cancers, results were consistent with those observed in the main analysis (Online Resource 6).

## Discussion

To the best of our knowledge, the present study is the first to investigate the longitudinal association between ID and low-grade chronic inflammation in a large cohort of relatively healthy European community-dwelling older adults.

At baseline, ID defined by sTfR levels was cross-sectionally associated with higher levels of hs-CRP and IL-6. Across all time points, the change from baseline in hs-CRP levels did not differ according to the iron status, while a significantly greater increase in IL-6 levels was observed in participants with ID at baseline. Participants with ID at any yearly time point over the follow-up had higher levels of inflammatory biomarkers at baseline. Similarly, we observed significant increases in hs-CRP levels and in IL-6 levels at follow-up time points when participants had ID at that time point.

Our results are partly consistent with those of a recent cross-sectional study. [20] Askar et al. found that CRP and IL-6 levels significantly increased in 20 middle-aged women with ID anemia (defined by ferritin < 15 μg/L and hemoglobin level of < 11.5 g/dL), when compared to their disease-free counterparts. Nevertheless, in our large study population of older adults, we did not find any statistically significant interaction between ID and baseline anemia, suggesting the results herein are independent of anemic status.

While the presence of ID at any yearly time point over the follow-up was associated with high levels of inflammatory biomarkers, we found opposite trends in non-ID participants. We observed a significant decrease in hs-CRP levels while IL-6 significantly increased over time. These different trends in inflammatory biomarkers levels were also observed in another large 3-year observational study including 4979 older adults, regardless of the iron status. [47] Since IL-6 is known to induce the production of CRP, this lack of timely overlap between IL-6 and hs-CRP levels could be explained by the intervention of hepcidin [48] and other cytokines not measured in the present study (e.g., IL-1, IL-17). [49] More recently, a review described that elevated IL-6 levels may also indicate protection, preservation, and/or repair of somatic tissue in an aging organism. [5].

Additionally, we found that baseline ID was associated with a significantly greater increase in IL-6 levels across all time points. These results may suggest ID as a promotor of low-grade chronic inflammation measured with IL-6. Given limited current scientific evidence on ID predicting inflammation and the observational nature of our study, our results are hypothesis generating. Thus, this work constitutes a first step to further investigate the role of ID and other regulators of iron on inflammation homeostasis such as hepcidin and their implications in healthy aging.

Furthermore, there is currently no consensus to define ID in older adults. We conducted sensitivity analyses to consider the most used definitions in clinical practice. Compared to our main analysis, contradictory results were obtained when using ferritin levels to define ID. Since ferritin is influenced by inflammatory conditions due to its additional function as acute-phase protein [50], our results confirmed that it might be less suitable to investigate ID in older adults. [51] Another point that has to be mentioned is the absence of evidence-based clinical thresholds to draw conclusions about the clinical relevance of increases or decreases in IL-6 and hs-CRP levels in our study population. For hs-CRP levels, a previous longitudinal large study used a cut-off of more than 3 mg/L to define the presence of systemic inflammation, since mildly elevated hs-CRP levels are particularly common in older adults. [52] When using this cut-off, it confirmed that our participants with ID at baseline or at any yearly follow-up time point presented a systemic inflammation since their hs-CRP levels were continually over 3 mg/L, while not in non-iron-deficient participants. Concerning IL-6 levels, current literature stated that IL-6 serum levels higher than 2 ng/L can be considered to define chronic inflammation in an older adult population. [53, 54] In the DO-HEALTH participants, IL-6 levels were higher than 2 ng/L at baseline and over time, regardless of the iron status. Nevertheless, the presence of ID over the follow-up was associated with higher increases of inflammatory biomarkers over time, compounding pre-existing chronic inflammation. Describing and interpreting the variations in inflammatory biomarkers over time could be of importance, especially when considering the concepts of aging and inflammaging. As evidenced by current literature, permanently elevated levels of IL-6 and CRP have been suggested as promoters of accelerated aging [10], frailty, decline in cognitive functions, age-related chronic disease, and a higher risk of mortality. [38] Hence, screening older adults for ID may be relevant for healthy aging, and preventing chronic diseases, and loss of autonomy in an increasingly, aging population. Once ID has been identified, education on dietary sources of iron as well as substances inhibiting iron absorption should be provided. [30] Alternatively, iron supplementation might be discussed. In octogenarians, low-dose supplementation of 15 mg of oral iron per day was reported to be safe and effective [55].

### Strengths and limitations

Our study took advantage of data collected in the DO-HEALTH trial, the largest European study on aging that included and followed 2157 community-dwelling older adults over 3 years. We assessed long-term chronic inflammation with annually repeated measurements of two reliable inflammatory biomarkers, broadly used in clinical practice. Besides, we extensively investigated the influence of ID on inflammatory biomarkers levels using four different definitions. We also strengthened the validity of our results in adjusting on a wide range of potential confounders and in performing several sensitivity analyses. However, a few limitations need consideration. Due to the observational nature of our study and despite adjustment for several relevant sociodemographic and clinical factors, we cannot exclude the possibility that the observed associations between ID and high levels of inflammatory biomarkers may be explained in part by residual confounding.

## Conclusion

Our results suggest that ID may play a relevant role in chronic low-grade inflammation measured by hs-CRP and IL-6 levels among relatively healthy older adults.

## Supplementary Information

Below is the link to the electronic supplementary material.

Supplementary file1 (DOCX 53 KB)

## Data Availability

In a first step, no data will be made available to researchers external to DO-HEALTH Research Group to allow primary researchers to fully exploit the dataset. The data will be shared in a second step according to a controlled access system.

## References

[1] Vetrano DL, Zucchelli A, Marconi E (2020). Predictors of iron-deficiency anemia in primary care older adults: a real-world European multi-country longitudinal study. Aging Clin Exp Res.

[2] Pasricha SR, Drakesmith H, Black J (2013). Control of iron deficiency anemia in low- and middle-income countries. Blood.

[3] Kassebaum NJ (2016) The global burden of Anemia. Hematol Oncol Clin North Am 30:247–308. 10.1016/j.hoc.2015.11.002 10.1016/j.hoc.2015.11.002 27040955

[4] Jonker FAM, van Hensbroek MB (2014) Anaemia, iron deficiency and susceptibility to infections. J Infect 69:S23–S27. 10.1016/j.jinf.2014.08.007 10.1016/j.jinf.2014.08.007 25264159

[5] Del Giudice M, Gangestad SW (2018). Rethinking IL-6 and CRP: Why they are more than inflammatory biomarkers, and why it matters. Brain Behav Immun.

[6] Nemeth E, Rivera S, Gabayan V (2004). IL-6 mediates hypoferremia of inflammation by inducing the synthesis of the iron regulatory hormone hepcidin. J Clin Investig.

[7] Cappellini MD, Comin-Colet J, de Francisco A (2017). Iron deficiency across chronic inflammatory conditions: International expert opinion on definition, diagnosis, and management. Am J Hematol.

[8] Nairz M, Theurl I, Wolf D (2016). Iron deficiency or anemia of inflammation? : Differential diagnosis and mechanisms of anemia of inflammation. Wien Med Wochenschr.

[9] Milan-Mattos JC, Anibal FF, Perseguini NM (2019). Effects of natural aging and gender on pro-inflammatory markers. Braz J Med Biol Res.

[10] Tsuboi A, Watanabe M, Kazumi T (2013). Association of low serum iron levels with low-grade inflammation and hyperadiponectinemia in community-living elderly women. J Atheroscler Thromb.

[11] Ferrucci L, Fabbri E (2018). Inflammageing: chronic inflammation in ageing, cardiovascular disease, and frailty. Nat Rev Cardiol.

[12] De Franceschi L, Iolascon A, Taher A (2017). Clinical management of iron deficiency anemia in adults: Systemic review on advances in diagnosis and treatment. Eur J Intern Med.

[13] Velissaris D, Pantzaris N, Koniari I (2017). C-reactive protein and frailty in the elderly: a literature review. J Clin Med Res.

[14] Rohrig G (2016). Anemia in the frail, elderly patient. Clin Interv Aging.

[15] Li Z-H, Zhong W-F, Lv Y-B (2019). Associations of plasma high-sensitivity C-reactive protein concentrations with all-cause and cause-specific mortality among middle-aged and elderly individuals. Immunity &amp; Ageing.

[16] Chen C, Liu Y, Cao Z (2019). Combined associations of hs-CRP and cognitive function with all-cause mortality among oldest-old adults in Chinese longevity areas: a prospective cohort study. Immunity &amp; Ageing.

[17] Ferrando-Martínez S, Romero-Sánchez MC, Solana R (2013). Thymic function failure and C-reactive protein levels are independent predictors of all-cause mortality in healthy elderly humans. Age.

[18] Giovannini S, Onder G, Liperoti R (2011). Interleukin-6, C-reactive protein, and tumor necrosis factor-alpha as predictors of mortality in frail, community-living elderly individuals. J Am Geriatr Soc.

[19] Robalo Nunes A, Fonseca C, Marques F, Belo A, Brilhante D, Cortez J (2017) Prevalence of anemia and iron deficiency in older Portuguese adults: An EMPIRE substudy. Geriatr Gerontol Int. 17:1814–1822. 10.1111/ggi.12966 10.1111/ggi.12966 28188967

[20] Askar S, Deveboynu SN, Er H (2019). Changes in pro-inflammatory cytokines and antimicrobial proteins in elderly women with iron deficiency anemia. Pak J Med Sci.

[21] Fairweather-Tait SJ, Wawer AA, Gillings R (2014). Iron status in the elderly. Mech Ageing Dev.

[22] Busti F, Campostrini N, Martinelli N (2014). Iron deficiency in the elderly population, revisited in the hepcidin era. Front Pharmacol.

[23] Bischoff-Ferrari HA, Molino C, Rival S (2020). DO-HEALTH: Vitamin D3 - Omega3 - Home exercise - Healthy aging and longevity trial - Design of a multinational clinical trial on healthy aging among European seniors. Contemp Clin Trials.

[24] Fullenbach C, Stein P, Glaser P (2020). Screening for iron deficiency in surgical patients based on noninvasive zinc protoporphyrin measurements. Transfusion.

[25] Joosten E, Van Loon R, Billen J (2002). Serum transferrin receptor in the evaluation of the iron status in elderly hospitalized patients with anemia. Am J Hematol.

[26] Choi CW, Cho WR, Park KH (2005). The cutoff value of serum ferritin for the diagnosis of iron deficiency in community-residing older persons. Ann Hematol.

[27] López-Sierra M, Calderón S, Gómez J (2012). Prevalence of anaemia and evaluation of transferrin receptor (sTfR) in the diagnosis of iron deficiency in the hospitalized elderly patients: anaemia clinical studies in chile. Anemia.

[28] Wang W, Knovich MA, Coffman LG (2010). Serum ferritin: Past, present and future. Biochim Biophys Acta.

[29] Guyatt GH, Patterson C, Ali M (1990). Diagnosis of iron-deficiency anemia in the elderly. Am J Med.

[30] Clenin GE (2017). The treatment of iron deficiency without anaemia (in otherwise healthy persons). Swiss Med Wkly.

[31] Yu D, Huo J, Xie L (2013). Meta-analysis of studies on cut-off value of serum ferritin for identifying iron deficiency. Wei Sheng Yan Jiu.

[32] Rimon E, Levy S, Sapir A (2002). Diagnosis of iron deficiency anemia in the elderly by transferrin receptor-ferritin index. Arch Intern Med.

[33] WHO (2011) Haemoglobin concentrations for the diagnosis of anaemia and assessment of severity. Geneva. https://apps.who.int/iris/handle/10665/85839

[34] Chocano-Bedoya PO, Bischoff-Ferrari HA; DO-HEALTH (2019) Vitamin D3-Omega-3-Home Exercise-Healthy Aging and Longevity Trial—Dietary Patterns in Five European Countries. In: Weaver CM, Bischoff-Ferrari H, Daly RM et al (eds) Nutritional Influences on Bone Health: 10th International Symposium. Springer International Publishing, pp 3–10

[35] Sangha O, Stucki G, Liang MH (2003). The Self-Administered Comorbidity Questionnaire: a new method to assess comorbidity for clinical and health services research. Arthritis Rheum.

[36] Fried LP, Tangen CM, Walston J (2001). Frailty in older adults: evidence for a phenotype. J Gerontol A Biol Sci Med Sci.

[37] Wolf AM, Hunter DJ, Colditz GA (1994). Reproducibility and validity of a self-administered physical activity questionnaire. Int J Epidemiol.

[38] Michaud M, Balardy L, Moulis G (2013). Proinflammatory cytokines, aging, and age-related diseases. J Am Med Dir Assoc.

[39] Wang JL, Shaw NS (2005). Iron status of the Taiwanese elderly: the prevalence of iron deficiency and elevated iron stores. Asia Pac J Clin Nutr.

[40] Seitz HK, Bataller R, Cortez-Pinto H (2018). Alcoholic liver disease. Nat Rev Dis Primers.

[41] Khan NA, Lawyer G, McDonough S (2020). Systemic biomarkers of inflammation, oxidative stress and tissue injury and repair among waterpipe, cigarette and dual tobacco smokers. Tob Control.

[42] WHO (2019) C-reactive protein concentrations as a marker of inflammation or infection for interpreting biomarkers of micronutrient status. Geneva

[43] Gkamprela E, Deutsch M, Pectasides D (2017). Iron deficiency anemia in chronic liver disease: etiopathogenesis, diagnosis and treatment. Ann Gastroenterol.

[44] Stein J, Connor S, Virgin G (2016). Anemia and iron deficiency in gastrointestinal and liver conditions. World J Gastroenterol.

[45] Batchelor EK, Kapitsinou P, Pergola PE (2020). Iron Deficiency in chronic kidney disease: updates on pathophysiology, diagnosis, and treatment. J Am Soc Nephrol.

[46] Gafter-Gvili A, Schechter A, Rozen-Zvi B (2019). Iron deficiency anemia in chronic kidney disease. Acta Haematol.

[47] Puzianowska-Kuźnicka M, Owczarz M, Wieczorowska-Tobis K (2016). Interleukin-6 and C-reactive protein, successful aging, and mortality: the PolSenior study. Immunity &amp; ageing : I &amp; A.

[48] Wrighting DM, Andrews NC (2006). Interleukin-6 induces hepcidin expression through STAT3. Blood.

[49] Eklund CM (2009) Proinflammatory cytokines in CRP baseline regulation. Adv Clin Chem 48:111–136. 10.1016/s0065-2423(09)48005-3 10.1016/s0065-2423(09)48005-3 19803417

[50] Thachil J (2016). The beneficial effect of acute phase increase in serum ferritin. Eur J Intern Med.

[51] Cankurtaran M, Yavuz BB, Halil M (2012). Increased ferritin levels could reflect ongoing aging-associated inflammation and may obscure underlying iron deficiency in the geriatric population. European Geriatric Medicine.

[52] Lassale C, Batty GD, Steptoe A (2019). Association of 10-year C-reactive protein trajectories with markers of healthy aging: findings from the english longitudinal study of aging. J Gerontol A Biol Sci Med Sci.

[53] Wyczalkowska-Tomasik A, Czarkowska-Paczek B, Zielenkiewicz M (2016). Inflammatory markers change with age, but do not fall beyond reported normal ranges. Arch Immunol Ther Exp (Warsz).

[54] Akbaraly TN, Hamer M, Ferrie JE (2013). Chronic inflammation as a determinant of future aging phenotypes. CMAJ.

[55] Rimon E, Kagansky N, Kagansky M (2005). Are we giving too much iron? Low-dose iron therapy is effective in octogenarians. Am J Med.

