# Supplementary material for: Iron deficiency and biomarkers of inflammation: a 3-year prospective analysis of the DO-HEALTH trial
Source: Aging Clin Exp Res. 2021 Sep 17;34(3):515–25. doi: 10.1007/s40520-021-01955-3 (PMC8894209; doi:10.1007/s40520-021-01955-3)
Supplement: Supplementary file 1 — Supplementary file1 (DOCX 53 KB) [file 40520_2021_1955_MOESM1_ESM.docx]

Supplementary Information

**Iron deficiency and biomarkers of inflammation: a three-year prospective analysis of the DO-HEALTH trial**

Maud Wieczorek, PhD; Franziska Schwarz; Angélique Sadlon, MD; Lauren A. Abderhalden, PhD; Caroline de Godoi Rezende Costa Molino, PhD; Donat R. Spahn, MD, Prof; Dominik J. Schaer, MD, Prof.; E. John Orav, PhD; Andreas Egli, MD; Heike A. Bischoff-Ferrari, MD, DrPH, Prof., for the DO-HEALTH Research group

**Corresponding author:**

Heike A. Bischoff-Ferrari, MD, DrPH

ORCID 0000-0003-0264-6318

University Hospital Zurich, Department of Aging Medicine and Aging Research,

Raemistrasse 101, 8091 Zurich, Switzerland

Phone: +41-44-255-2757, email: [Heike.Bischoff@usz.ch](mailto:Heike.Bischoff@usz.ch)

[DO-HEALTH Research Group 2](#_Toc78201351)

[Online Resource 1. Changes from baseline in hs-CRP by baseline iron status, defined with soluble transferrin receptor levels by sex 4](#_Toc78201352)

[Online Resource 2. Changes from baseline in IL-6 levels by baseline iron status defined with soluble transferrin receptor levels by age group 5](#_Toc78201353)

[Online Resource 3. Changes in hs-CRP and IL-6 levels by baseline iron status defined with ferritin levels and the transferrin-ferritin index 6](#_Toc78201354)

[Online Resource 4. Changes from baseline in hs-CRP and IL-6 levels by iron status at each yearly time point over the follow-up, defined with ferritin levels and the transferrin-ferritin index 8](#_Toc78201355)

[Online Resource 5. Changes from baseline in hs-CRP and IL-6 levels by recurrence of iron deficiency 11](#_Toc78201356)

[Online Resource 6. Changes from baseline in hs-CRP and IL-6 levels by iron status at baseline and at each yearly time point over the follow-up, defined with soluble transferrin receptor levels when excluding participants who reported kidney disease and liver disease at baseline and participants with incident invasive cancer over the follow-up 12](#_Toc78201357)

This appendix has been provided by the authors to give readers additional information about DO-HEALTH Research Group.

# DO-HEALTH Research Group

**DO-HEALTH Consortium**

(in bold: Governing Board members; in bold and underlined: Chair; underlined: Team members).

**Prof Heike A Bischoff-Ferrari MD**, DO-HEALTH Coordinator, Principal Investigator and Zurich Site Investigator, leads all endpoints analyses and co-leads the studies ‘DO-HEALTH health economic model’, ‘novel biomarkers of immunity’, ‘novel biomarkers of muscle and bone communication’, University Hospital Zurich, University of Zurich and Waid City Hospital, Zurich, Switzerland, Andreas Egli MD, Sandrine Rival PhD.

**Prof Bruno Vellas MD**, Toulouse Site Investigator, contributes to the primary endpoint cognitive decline, and Sophie Guyonnet PhD, CHU Toulouse and University of Toulouse III, Toulouse, France.

**Prof René Rizzoli MD**, Geneva Site Investigator, contributes to all bone and muscle related endpoints and explores the contribution of protein intake to the benefit of the interventions, Emmanuel Biver MD, and Fanny Merminod RD, Geneva University Hospitals and Faculty of Medicine, Geneva, Switzerland.

**Prof Reto W Kressig MD**, Basel Site Investigator, contributes to gait analyses and dual task assessments, and Stephanie Bridenbaugh MD, University Department of Geriatric Medicine FELIX PLATTER and University of Basel, Basel, Switzerland. Prof. Norbert Suhm, Dept. of Traumatology, University Hospital Basel, contributes to fracture healing study DO-HEALTH.

**Prof José A P Da Silva MD**, Coimbra Site Investigator, explores the treatment effects on vertebral fractures, and musculoskeletal pain and function, Centro Hospitalar e Universitário de Coimbra, and Faculty of Medicine, University of Coimbra, Coimbra, Portugal, Cátia CM Duarte MD, Centro Hospitalar e Universitário de Coimbra, Coimbra, Portugal, and Ana Filipa Pinto RN, Faculty of Medicine, University of Coimbra, Coimbra, Portugal.

**Prof Dieter Felsenberg MD**, Berlin Site Investigator, performs the central DO-HEALTH DEXA quality control and evaluation of DEXA measurements, Hendrikje Börst Dipl.Wiss-org, and Gabriele Armbrecht MD, Charité Universitätsmedizin Berlin, Berlin, Germany.

**Prof Michael Blauth MD**, Innsbruck Site Investigator, explores the functionality after fracture, and Anna Spicher MD, Medical University of Innsbruck, Innsbruck, Austria.

**Prof David T Felson MD**, co-leads ‘DO-HEALTH osteoarthritis study’, Manchester Academic Health Science Centre, Manchester, United Kingdom and Boston University School of Medicine, Boston, MA, USA.

**Prof John A Kanis MD** leads the study ‘contribution of fall risk to absolute fracture risk within the FRAX model’, University of Sheffield Medical School, Sheffield, United Kingdom and Australian Catholic University, Melbourne, Victoria, Australia. Prof Eugene V Mccloskey MD, co-leads the study ‘contribution of fall risk to absolute fracture risk within the FRAX model’, University of Sheffield, Sheffield, United Kingdom, and Elena Johansson MD, University of Sheffield Medical School, Sheffield, United Kingdom and Catholic University of Australia, Melbourne, Victoria, Australia.

**Prof Bernhard Watzl PhD**, co-leads the study ‘novel biomarkers of immunity’, Manuel Rodriguez Gomez PhD, Max Rubner-Institut, Karlsruhe, Germany.

**Prof Lorenz Hofbauer MD**, co-leads the study ‘novel biomarkers of muscle and bone communication’, FOÄ Dr. Elena Tsourdi, and Professor Martina Rauner PhD, Dresden University Medical Center and Center for Regenerative Therapies Dresden, Dresden, Germany.

**Uwe Siebert MD**, co-leads the study ‘DO-HEALTH health economic model’, UMIT - University for Health Sciences, Medical Informatics and Technology, Hall i.T., Austria and Harvard T.H. Chan School of Public Health, Boston, MA, USA and Massachusetts General Hospital, Harvard Medical School, Boston, MA, USA.

**Prof John A Kanis MD**, leads DO-HEALTH impact and communication of osteoporosis-related findings on a broad level, and Philippe Halbout PhD, IOF.

**Stephen M Ferrari**, leads DO-HEALTH software development (Electronic Data Capture system and interactive practical software for seniors and health care professionals that teaches main findings of DO-HEALTH), Ferrari Data Solutions, Feldmeilen, Switzerland.

**Benno Gut**, leads DO-HEALTH visual communication (SHEP avatar) and DO-HEALTH corporate design structures (logo, website software and communication tools), gut pictures, Horgen, Switzerland.

**Marième Ba**, was the DO-HEALTH independent clinical monitoring partner, Pharmalys, Borehamwood, United Kingdom.

**Jonas Wittwer Schegg PhD**, industrial partner representative bringing expertise and facilities in plasma analytics for 25-Hydroxyvitamin D and Omega-3 Fatty Acids and providing the study medication (Vitamin D, Omega-3 fatty acids), and Stéphane Etheve, DSM Nutritional Products, Kaiseraugst, Switzerland, and Manfred Eggersdorfer PhD, University Medical Center Groningen, Gronigen, The Netherlands.

**Carla Sofia Delannoy PhD**, industrial partner representative providing financial support to DO-HEALTH central coordination, Nestlé Health Science, Lausanne, Switzerland.

**Monika Reuschling PhD**, industrial partner representative providing assays for the large DO-HEALTH biomarker study to define reference ranges of common biomarkers in adults age 70+, Roche diagnostiscs, Rotkreuz, Switzerland.

**DO-HEALTH Scientific Advisory Board members and collaborators on specific outcomes**

**Prof Endel J Orav PhD** (Head Biostatistician), Harvard T.H. Chan School of Public Health, Boston, MA, USA.

**Prof Walter C Willett MD** (CVD, Cancer, Omega-3, FFQ), Harvard T H Chan School of Public Health, Boston, MA, USA.

**Prof JoAnn E Manson MD** (PI VITAL, CVD, Diabetes), Brigham and Women's Hospital, Harvard Medical School, Boston, MA, USA.

**Prof Bess Dawson-Hughes MD** (Fractures, Falls, Vitamin D), Tufts University, Boston, MA, USA.

**Prof Hannes B Staehelin MD** (Cognition, Function), University of Basel, Basel, Switzerland.

**Prof Paul W Walter** (Nutrition – glucose metabolism), University of Basel, Basel, Switzerland.

**Prof. Walter Dick** (Fractures, Osteoarthritis), University of Basel, Basel, Switzerland.

**Prof Michael Fried MD** (Gastro-Intestinal health), University of Zurich, Zurich, Switzerland.

**Prof Arnold von Eckardstein MD** (Biomarkers reference values), University of Zurich, Zurich, Switzerland.

**Prof Robert Theiler MD** (Falls, Osteoarthritis, DO-HEALTH Exercise program)**,** University Hospital Zurich and University of Zurich, Zurich, Switzerland.

**Prof Hans-Peter Simmen MD** (Traumatology), University of Zurich, Zurich, Switzerland.

**Prof Wolfgang Langhans PhD** (Nutrition – Diabetes), ETH Zurich, Zurich, Switzerland.

**Prof Annelies Zinkernagel MD** (Infections – bacterial), University Hospital of Zurich, Zurich, Switzerland.

**Prof Nicolas Mueller MD** (Infections – viral), University Hospital of Zurich, Zurich, Switzerland.

**Prof Oliver Distler MD** (Inflammatory Arthritis), University Hospital of Zurich, Zurich, Switzerland.

**Prof Klaus Graetz MD** (Oral/Dental Health), University Hospital of Zurich, Zurich, Switzerland.

**Prof Ina Nitschke MD** (Dental Health), University Hospital of Zurich, Zurich, Switzerland.

**Prof. Thomas Dietrich** (Oral Health), University of Birmingham, UK.

**Prof Walter Baer MD** (Mortality), University of Zurich, Zurich, Switzerland.

**Prof Klara Landau MD (**Visual Acuity**)**, University Hospital of Zurich, Zurich, Switzerland.

**Prof Frank Ruschitzka MD** (Cardiology), University Hospital of Zurich, Zurich, Switzerland.

**Prof Markus Manz MD** (Hematology), University Hospital of Zurich, Zurich, Switzerland.

**Prof Peter Burckhardt MD** (Calcium intake, Metabolism), University of Lausanne, Lausanne, Switzerland.

**🞳** In Memory of  Dieter Felsenberg, a passionate scientist in clinical muscle and bone research

# Online Resource 1. Changes from baseline in hs-CRP by baseline iron status, defined with soluble transferrin receptor levels by sex

|  | **Baseline iron deficiency**  sTfR levels  > 28.1 nmol/L | **No baseline iron deficiency**  sTfR levels  ≤ 28.1 nmol/L | Mean difference in change from baseline (95% CI) | P value for mean difference in change from baseline between groups ^a^ |
| --- | --- | --- | --- | --- |
| **hs-CRP (mg/L)** |  |  |  |  |
| **Male participants** | n=210 | n=614 |  |  |
| Unadjusted at baseline, mean (SD) | 3.96 (0.66) | 2.52 (0.20) | **1.45 (0.09 to 2.81)** | **.04** |
| Adjusted change at Year 1 (95% CI) | -0.12 (-0.82 to 0.58) | -0.27 (-0.71 to 0.16) | 0.15 (-0.68 to 0.98) | .72 |
| Adjusted change at Year 2 (95% CI) | -0.34 (-0.88 to 0.21) | 0.01 (-0.74 to 0.76) | -0.35 (-1.28 to 0.58) | .46 |
| Adjusted change at Year 3 (95% CI) | **-0.48 (-0.90 to -0.07)** | -0.16 (-0.62 to 0.30) | -0.32 (-0.94 to 0.29) | .31 |
| Adjusted change across all time points (95% CI) | -0.31 (-0.72 to 0.09) | -0.14 (-0.49 to 0.21) | -0.17 (-0.71 to 0.37) | .53 |
| **Female participants** | n=363 | n=954 |  |  |
| Unadjusted at baseline, mean (SD) | 4.00 (0.39) | 2.48 (0.11) | **1.52 (0.72 to 2.33)** | **<.001** |
| Adjusted change at Year 1 (95% CI) | 0.13 (-0.43 to 0.69) | -0.28 (-0.58 to 0.01) | 0.41 (-0.24 to 1.07) | .21 |
| Adjusted change at Year 2 (95% CI) | 0.44 (-0.23 to 1.11) | 0.01 (-0.29 to 0.31) | 0.43 (-0.30 to 1.17) | .25 |
| Adjusted change at Year 3 (95% CI) | -0.15 (-0.56 to 0.27) | -0.30 (-0.70 to 0.10) | 0.16 (-0.43 to 0.75) | .60 |
| Adjusted change across all time points (95% CI) | 0.14 (-0.21 to 0.50) | -0.19 (-0.41 to 0.03) | 0.34 (-0.10 to 0.77) | .13 |

^a^ p-values correspond to the mean differences in biomarkers levels or changes in biomarker levels between iron deficient and non-iron deficient groups. Baseline levels are compared using a t-test. Yearly changes from baseline are compared by repeated measures linear regression with interaction terms between iron deficiency status and time. Overall differences across all time points are compared by repeated measures linear regression with a main effect for iron deficiency.

Models are adjusted for treatment allocation, age, sex, center, body mass index over the follow-up, alcohol consumption, tobacco consumption, polypharmacy, number of comorbidities, frailty status (pre-frailty), frequency of physical activity, yearly incidence rate of infections, and baseline level of the outcome.

sTfR: soluble Transferrin Receptor

Values in bold indicate significant P-values

# Online Resource 2. Changes from baseline in IL-6 levels by baseline iron status defined with soluble transferrin receptor levels by age group

|  | **Baseline iron deficiency**  sTfR levels  > 28.1 nmol/L | **No baseline iron deficiency**  sTfR levels  ≤ 28.1 nmol/L | Mean difference in change from baseline (95% CI) | P value for mean difference in change from baseline between groups ^a^ |
| --- | --- | --- | --- | --- |
| **IL-6 (ng/L)** |  |  |  |  |
| **Age < 75** | n=285 | n=942 |  |  |
| Unadjusted at baseline, mean (SD) | 3.95 (0.26) | 3.19 (0.21) | **0.76 (0.09 to 1.43)** | **.03** |
| Adjusted change at Year 1 (95% CI) | **0.60 (0.21 to 0.99)** | **0.56 (0.26 to 0.86)** | 0.04 (-0.47 to 0.55) | .87 |
| Adjusted change at Year 2 (95% CI) | **0.72 (0.34 to 1.11)** | **0.79 (0.47 to 1.10)** | -0.06 (-0.58 to 0.45) | .81 |
| Adjusted change at Year 3 (95% CI) | **0.48 (0.09 to 0.88)** | **0.42 (0.16 to 0.69)** | 0.06 (-0.44 to 0.55) | .82 |
| Adjusted change across all time points (95% CI) | **0.60 (0.31 to 0.90)** | **0.59 (0.37 to 0.81)** | 0.01 (-0.38 to 0.40) | .95 |
| **Age ≥ 75** | n=288 | n=626 |  |  |
| Unadjusted at baseline, mean (SD) | 5.49 (0.72) | 3.88 (0.17) | **1.61 (0.15 to 3.07)** | **.03** |
| Adjusted change at Year 1 (95% CI) | 1.22 (0.34 to 2.10) | 0.24 (-0.19 to 0.68) | 0.98 (-0.00 to 1.96) | .05 |
| Adjusted change at Year 2 (95% CI) | 1.20 (0.34 to 2.07) | 0.56 (0.09 to 1.03) | 0.64 (-0.38 to 1.66) | .22 |
| Adjusted change at Year 3 (95% CI) | 1.86 (0.64 to 3.07) | 0.51 (0.12 to 0.90) | **1.35 (0.05 to 2.64)** | **.04** |
| Adjusted change across all time points (95% CI) | 1.43 (0.75 to 2.11) | 0.44 (0.13 to 0.75) | **0.99 (0.21 to 1.77)** | **.01** |

^a^ p-values correspond to the mean differences in biomarkers levels or changes in biomarker levels between iron deficient and non-iron deficient groups. Baseline levels are compared using a t-test. Yearly changes from baseline are compared by repeated measures linear regression with interaction terms between iron deficiency status and time. Overall differences across all time points are compared by repeated measures linear regression with a main effect for iron deficiency.

Models are adjusted for treatment allocation, age, sex, center, body mass index over the follow-up, alcohol consumption, tobacco consumption, polypharmacy, number of comorbidities, frailty status (pre-frailty), frequency of physical activity, yearly incidence rate of infections, and baseline level of the outcome.

sTfR: soluble Transferrin Receptor

Values in bold indicate significant P-values

# Online Resource 3. Changes in hs-CRP and IL-6 levels by baseline iron status defined with ferritin levels and the transferrin-ferritin index

|  | **Baseline iron deficiency**  Ferritin levels < 45 μg/L | **No baseline iron deficiency**  Ferritin levels ≥ 45 μg/L | Mean difference in change from baseline (95% CI) | P value for mean difference in change from baseline between groups ^a^ |
| --- | --- | --- | --- | --- |
|  | n=199 | n=1942 |  |  |
| **hs-CRP (mg/L)** |  |  |  |  |
| Unadjusted at baseline, mean (SD) | 2.06 (0.14) | 2.98 (0.13) | **-0.92 (-1.30 to -0.53)** | **<.001** |
| Adjusted change at Year 1 (95% CI) | -0.29 (-0.81 to 0.24) | -0.19 (-0.42 to 0.04) | -0.09 (-0.66 to 0.47) | .74 |
| Adjusted change at Year 2 (95% CI) | -0.27 (-0.78 to 0.25) | 0.08 (-0.22 to 0.39) | -0.35 (-0.92 to 0.21) | .22 |
| Adjusted change at Year 3 (95% CI) | -0.56 (-0.98 to -0.14) | -0.23 (-0.48 to 0.03) | -0.33 (-0.82 to 0.15) | .18 |
| Adjusted change across all time points (95% CI) | -0.37 (-0.73 to -0.01) | -0.11 (-0.28 to 0.05) | -0.26 (-0.63 to 0.11) | .17 |
| **IL-6 (ng/L)** |  |  |  |  |
| Unadjusted at baseline, mean (SD) | 3.47 (0.20) | 3.83 (0.16) | -0.36 (-0.86 to 0.14) | .16 |
| Adjusted change at Year 1 (95% CI) | 0.94 (-0.06 to 1.95) | **0.52 (0.29 to 0.75)** | 0.42 (-0.59 to 1.44) | .41 |
| Adjusted change at Year 2 (95% CI) | **0.82 (0.35 to 1.29)** | **0.77 (0.53 to 1.01)** | 0.05 (-0.46 to 0.56) | .85 |
| Adjusted change at Year 3 (95% CI) | 1.03 (0.17 to 1.89) | **0.60 (0.37 to 0.84)** | 0.43 (-0.46 to 1.31) | .34 |
| Adjusted change across all time points (95% CI) | 0.93 (0.40 to 1.46) | **0.63 (0.46 to 0.80)** | 0.30 (-0.23 to 0.83) | .27 |
|  | **Baseline iron deficiency**  Ferritin levels < 30 μg/L | **No baseline iron deficiency**  Ferritin levels ≥ 30 μg/L | Mean difference in change from baseline (95% CI) | P value for mean difference in change from baseline between groups ^a^ |
|  | n=90 | n=2051 |  |  |
| **hs-CRP (mg/L)** |  |  |  |  |
| Unadjusted at baseline, mean (SD) | 2.00 (0.19) | 2.93 (0.13) | **-0.93 (-1.38 to -0.49)** | **<.001** |
| Adjusted change at Year 1 (95% CI) | -0.27 (-1.18 to 0.65) | -0.20 (-0.41 to 0.02) | -0.07 (-1.01 to 0.86) | .88 |
| Adjusted change at Year 2 (95% CI) | -0.34 (-1.11 to 0.44) | 0.07 (-0.23 to 0.37) | -0.41-(1.22 to 0.41) | .33 |
| Adjusted change at Year 3 (95% CI) | **-0.64 (-1.24 to -0.03)** | -0.24 (-0.48 to 0.00) | -0.39 (-1.05 to 0.26) | .24 |
| Adjusted change across all time points (95% CI) | -0.41 (-0.99 to 0.17) | -0.12 (-0.28 to 0.04) | -0.29 (-0.88 to 0.30) | .33 |
| **IL-6 (ng/L)** |  |  |  |  |
| Unadjusted at baseline, mean (SD) | 3.48 (0.24) | 3.81 (0.16) | -0.33 (-0.90 to 0.23) | .25 |
| Adjusted change at Year 1 (95% CI) | 1.49 (-0.55 to 3.53) | **0.52 (0.29 to 0.74)** | 0.97 (1.07 to 3.02) | .35 |
| Adjusted change at Year 2 (95% CI) | **0.75 (0.12 to 1.39)** | **0.77 (0.54 to 1.00)** | -0.02 (-0.68 to 0.64) | .96 |
| Adjusted change at Year 3 (95% CI) | **0.77 (0.04 to 1.49)** | **0.64 (0.40 to 0.87)** | 0.13 (-0.63 to 0.89) | .74 |
| Adjusted change across all time points (95% CI) | **1.00 (0.20 to 1.80)** | **0.64 (0.48 to 0.81)** | 0.36 (-0.44 to 1.17) | .38 |
|  | **Baseline iron deficiency**  sTfR-Ferritin index > 1.5 | **No baseline iron deficiency**  sTfR-Ferritin index ≤ 1.5 | Mean difference in change from baseline (95% CI) | P value for mean difference in change from baseline between groups ^a^ |
|  | n=755 | n=1386 |  |  |
| **hs-CRP (mg/L)** |  |  |  |  |
| Unadjusted at baseline, mean (SD) | 3.35 (0.23) | 2.64 (0.14) | **0.71 (0.18 to 1.24)** | **.009** |
| Adjusted change at Year 1 (95% CI) | 0.29 (-0.15 to 0.73) | **-0.46 (-0.68 to -0.23)** | **0.75 (0.25 to 1.24)** | **.003** |
| Adjusted change at Year 2 (95% CI) | 0.34 (-0.12 to 0.80) | -0.10 (-0.46 to 0.26) | 0.44 (-0.13 to 1.00) | .13 |
| Adjusted change at Year 3 (95% CI) | -0.13 (-0.55 to 0.30) | -0.33 (-0.60 to -0.05) | 0.20 (-0.31 to 0.71) | .44 |
| Adjusted change across all time points (95% CI) | 0.17 (-0.12 to 0.46) | **-0.29 (-0.47 to -0.12)** | **0.46 (0.13 to 0.79)** | **.006** |
| **IL-6 (ng/L)** |  |  |  |  |
| Unadjusted at baseline, mean (SD) | 4.38 (0.31) | 3.48 (0.16) | **0.91 (0.23 to 1.58)** | **.009** |
| Adjusted change at Year 1 (95% CI) | **0.90 (0.46 to 1.34)** | **0.38 (0.11 to 0.65)** | **0.53 (0.00 to 1.05)** | **.05** |
| Adjusted change at Year 2 (95% CI) | **1.05 (0.65 to 1.44)** | **0.63 (0.33 to 0.92)** | 0.42 ( -0.10 to 0.94) | .11 |
| Adjusted change at Year 3 (95% CI) | **0.89 (0.38 to 1.40)** | **0.51 (0.25 to 0.77)** | 0.38 (-0.22 to 0.97) | .21 |
| Adjusted change across all time points (95% CI) | **0.95 (0.64 to 1.26)** | **0.50 ( 0.29 to 0.72)** | **0.44 (0.04 to 0.85)** | **.03** |

^a^ p-values correspond to the mean differences in biomarkers levels or changes in biomarker levels between iron deficient and non-iron deficient groups. Baseline levels are compared using a t-test. Yearly changes from baseline are compared by repeated measures linear regression with interaction terms between iron deficiency status and time. Overall differences across all time points are compared by repeated measures linear regression with a main effect for iron deficiency.

Models are adjusted for treatment allocation, age, sex, center, body mass index over the follow-up, alcohol consumption, tobacco consumption, polypharmacy, number of comorbidities, frailty status (pre-frailty), frequency of physical activity, yearly incidence rate of infections, and baseline level of the outcome.

sTfR: soluble Transferrin Receptor

Values in bold indicate significant P-values

# Online Resource 4. Changes from baseline in hs-CRP and IL-6 levels by iron status at each yearly time point over the follow-up, defined with ferritin levels and the transferrin-ferritin index

|  | **Iron deficiency at any yearly follow-up time point**  Ferritin levels < 45 μg/L | **No iron deficiency at any yearly follow-up time point** Ferritin levels ≥ 45 μg/L | Mean difference in change from baseline (95% CI) | P value for mean difference in change from baseline between groups ^a^ |
| --- | --- | --- | --- | --- |
| **hs-CRP (mg/L)** |  |  |  |  |
| Unadjusted at baseline, mean (SD) | 2.00 (0.14) | 2.98 (0.13) | **-0.97 (-1.35 to -0.59)** | **<.001** |
| Adjusted change at Year 1 (95% CI) | **-0.99 (-1.39 to -0.59)** [n=188] | -0.11 (-0.35 to 0.12) [n=1730] | **-0.88 (-1.36 to -0.39)** | **<.001** |
| Adjusted change at Year 2 (95% CI) | **-1.06 (-1.50 to -0.61)** [n=216] | 0.20 (-0.12 to 0.52) [n=1615] | **-1.26 (-1.80 to -0.71)** | **<.001** |
| Adjusted change at Year 3 (95% CI) | **-1.06 (-1.44 to -0.67)** [n=201] | -0.16 (-0.42 to 0.10) [n=1628] | **-0.90 (-1.38 to -0.42)** | **<.001** |
| Adjusted change across all time points (95% CI) | **-1.03 (-1.39 to -0.68)** | -0.02 (-0.19 to 0.15) | **-1.01 (-1.41 to -0.61)** | **<.001** |
| **IL-6 (ng/L)** |  |  |  |  |
| Unadjusted at baseline, mean (SD) | 3.29 (0.21) | 3.84 (0.16) | **-0.55 (-1.04 to -0.06)** | **.03** |
| Adjusted change at Year 1 (95% CI) | 0.75 (-0.34 to 1.84)  [n=188] | **0.54 (0.30 to 0.77)**  [n=1730] | 0.21 (-0.91 to 1.34) | .71 |
| Adjusted change at Year 2 (95% CI) | 0.29 (-0.22 to 0.80)  [n=216] | **0.83 (0.57 to 1.10)**  [n=1615] | -0.55 (-1.17 to 0.08) | .09 |
| Adjusted change at Year 3 (95% CI) | 0.77 (-0.05 to 1.60)  [n=201] | **0.63 (0.38 to 0.87)**  [n=1628] | 0.15 (-0.73 to 1.03) | .74 |
| Adjusted change across all time points (95% CI) | **0.61 (0.05 to 1.16)** | **0.67 (0.48 to 0.85)** | -0.06 (-0.67 to 0.55) | .85 |
|  | **Iron deficiency at any yearly follow-up time point**  Ferritin levels < 30 μg/L | **No iron deficiency at any yearly follow-up time point** Ferritin levels ≥ 30 μg/L | Mean difference in change from baseline (95% CI) | P value for mean difference in change from baseline between groups ^a^ |
| **hs-CRP (mg/L)** |  |  |  |  |
| Unadjusted at baseline, mean (SD) | 1.97 (0.17) | 2.93 (0.13) | **-0.96 (-1.37 to -0.55)** | **<.001** |
| Adjusted change at Year 1 (95% CI) | -1.06 (-1.55 to -0.57)  [n=86] | -0.16 (-0.38 to 0.06) [n=1832] | **-0.90 (-1.45 to -0.35)** | **.001** |
| Adjusted change at Year 2 (95% CI) | **-0.91 (-1.38 to -0.45)**  [n=95] | 0.10 (-0.20 to 0.41) [n=1736] | **-1.02 (-1.55 to -0.48)** | **<.001** |
| Adjusted change at Year 3 (95% CI) | **-0.96 (-1.48 to -0.44)** [n=105] | -0.22 (-0.46 to 0.03) [n=1724] | **-0.74 (-1.32 to -0.16)** | **.01** |
| Adjusted change across all time points (95% CI) | **-0.98 (-1.39 to -0.57)** | -0.09 (-0.26 to 0.08) | **-0.89 (-1.32 to -0.46)** | **<.001** |
| **IL-6 (ng/L)** |  |  |  |  |
| Unadjusted at baseline, mean (SD) | 3.14 (0.46) | 3.82 (0.15) | -0.68 (-1.62 to 0.26) | .16 |
| Adjusted change at Year 1 (95% CI) | 0.74 (-0.94 to 2.42)  [n=86] | **0.55 (0.30 to 0.80)**  [n=1832] | 0.19 (-1.57 to 1.94) | .83 |
| Adjusted change at Year 2 (95% CI) | 0.18 (-0.85 to 1.21)  [n=95] | **0.80 (0.56 to 1.05)**  [n=1736] | -0.62 (-1.74 to 0.49) | .27 |
| Adjusted change at Year 3 (95% CI) | 0.61 (-0.65 to 1.87)  [n=105] | **0.64 (0.40 to 0.89)**  [n=1724] | -0.03 (-1.36 to 1.30) | .96 |
| Adjusted change across all time points (95% CI) | 0.51 (-0.65 to 1.67) | **0.67 (0.48 to 0.85)** | -0.16 (-1.39 to 1.08) | .80 |
|  | **Iron deficiency at any yearly follow-up time point**  sTfR-Ferritin index > 1.5 | **No iron deficiency at any yearly follow-up time point** sTfR-Ferritin index ≤ 1.5 | Mean difference in change from baseline (95% CI) | P value for mean difference in change from baseline between groups ^a^ |
| **hs-CRP (mg/L)** |  |  |  |  |
| Unadjusted at baseline, mean (SD) | 3.28 (0.23) | 2.68 (0.14) | **0.61 (0.08 to 1.13)** | **.02** |
| Adjusted change at Year 1 (95% CI) | -0.20 (-0.60 to 0.19)  [n=675] | -0.17 (-0.50 to 0.17) [n=1225] | -0.04 (-0.63 to 0.56) | .90 |
| Adjusted change at Year 2 (95% CI) | 0.06 (-0.43 to 0.54)  [n=686] | 0.07 (-0.33 to 0.46)  [n=1145] | -0.01 (-0.67 to 0.64) | .97 |
| Adjusted change at Year 3 (95% CI) | -0.31 (-0.85 to 0.24)  [n=657] | -0.19 (-0.47 to 0.08)  [n=1100] | -0.11 (-0.77 to 0.55) | .74 |
| Adjusted change across all time points (95% CI) | -0.15 (-0.50 to 0.20) | -0.10 (-0.34 to 0.14) | -0.05 (-0.55 to 0.44) | .83 |
| **IL-6 (ng/L)** |  |  |  |  |
| Unadjusted at baseline, mean (SD) | 4.31 (0.28) | 3.51 (0.16) | **0.80 (0.19 to 1.40)** | **.01** |
| Adjusted change at Year 1 (95% CI) | **0.90 (0.46 to 1.34)**  [n=675] | **0.38 (0.08 to 0.68)**  [n=1225] | 0.52 (-0.04 to 1.09) | **.07** |
| Adjusted change at Year 2 (95% CI) | **1.20 (0.76 to 1.64)**  [n=686] | **0.52 (0.23 to 0.82)**  [n=1145] | **0.68 (0.11 to 1.25)** | **.02** |
| Adjusted change at Year 3 (95% CI) | **1.06 (0.52 to 1.60)**  [n=657] | **0.41 (0.16 to 0.66)**  [n=1100] | **0.65 (0.03 to 1.28)** | **.04** |
| Adjusted change across all time points (95% CI) | **1.05 (0.70 to 1.41)** | **0.44 (0.22 to 0.66)** | **0.62 (0.16 to 1.07)** | **.008** |

^a^ p-values correspond to the mean differences in biomarkers levels or changes in biomarker levels between iron deficient and non-iron deficient groups. Baseline levels are compared using a t-test. Yearly changes from baseline are compared by repeated measures linear regression with interaction terms between iron deficiency status and time. Overall differences across all time points are compared by repeated measures linear regression with a main effect for iron deficiency.

Models are adjusted for treatment allocation, age, sex, center, body mass index over the follow-up, alcohol consumption, tobacco consumption, polypharmacy, number of comorbidities, frailty status (pre-frailty), frequency of physical activity, yearly incidence rate of infections, baseline iron status and baseline level of the outcome.

Numbers between squared brackets indicate the number of iron-deficient and non-iron deficient participants at each yearly time point

sTfR: soluble Transferrin Receptor

Values in bold indicate significant P-values

# Online Resource 5. Changes from baseline in hs-CRP and IL-6 levels by recurrence of iron deficiency

|  | **Iron deficiency at 2 or more consecutive time points**  sTfR levels > 28.1 nmol/L | **Iron deficiency at 1 time point**  sTfR levels > 28.1 nmol/L | Mean difference in change from baseline (95% CI) | P value for mean difference in change from baseline between groups ^a^ |
| --- | --- | --- | --- | --- |
|  | n=473 | n=270 |  |  |
| **hs-CRP (mg/L)** |  |  |  |  |
| Unadjusted at baseline, mean (SD) | 3.63 (0.37) | 3.30 (0.43) | 0.32 (-0.79 to 1.44) | 0.57 |
| Adjusted change at Year 1 (95% CI) | 0.05 (-0.57 to 0.67) | -0.74 (-1.42 to -0.06) | 0.79 (-0.22 to 1.80) | 0.13 |
| Adjusted change at Year 2 (95% CI) | 0.30 (-0.34 to 0.95) | **-0.95 (-1.64 to -0.25)** | **1.25 (0.20 to 2.29)** | **0.02** |
| Adjusted change at Year 3 (95% CI) | 0.14 (-0.72 to 0.99) | -0.92 (-1.63 to -0.22) | 1.06 (-0.18 to 2.31) | 0.10 |
| Adjusted change across all time points (95% CI) | 0.16 (-0.37 to 0.70) | **-0.87 (-1.35 to -0.39)** | **1.03 (0.17 to 1.90)** | **0.02** |
| **IL-6 (ng/L)** |  |  |  |  |
| Unadjusted at baseline, mean (SD) | 4.54 (0.45) | 3.62 (0.26) | 0.92 (-0.10 to 1.95) | 0.08 |
| Adjusted change at Year 1 (95% CI) | **1.13 (0.57 to 1.69)** | 0.00 (-0.57 to 0.57) | **1.13 (0.28 to 1.98)** | **0.009** |
| Adjusted change at Year 2 (95% CI) | **1.33 (0.79 to 1.88)** | 0.31 (-0.22 to 0.84) | **1.03 (0.19 to 1.87)** | **0.02** |
| Adjusted change at Year 3 (95% CI) | **1.47 (0.72 to 2.21)** | 0.35 (-0.29 to 0.98) | **1.12 (0.05 to 2.18)** | **0.04** |
| Adjusted change across all time points (95% CI) | **1.31 (0.86 to 1.76)** | 0.22 (-0.24 to 0.68) | **1.09 (0.36 to 1.82)** | **0.004** |

^a^ p-values correspond to the mean differences in biomarkers levels or changes in biomarker levels between participants with recurrent iron deficiency and participants with iron deficiency at one time point. Baseline levels are compared using a t-test. Yearly changes from baseline are compared by repeated measures linear regression with interaction terms between iron deficiency status and time. Overall differences across all time points are compared by repeated measures linear regression with a main effect for iron deficiency.

Models are adjusted for treatment allocation, age, sex, center, body mass index over the follow-up, alcohol consumption, tobacco consumption, polypharmacy, number of comorbidities, frailty status (pre-frailty), frequency of physical activity, yearly incidence rate of infections, baseline iron status and baseline level of the outcome.

Numbers between squared brackets indicate the number of iron-deficient and non-iron deficient participants at each yearly time point

sTfR: soluble Transferrin Receptor

Values in bold indicate significant P-values

# Online Resource 6. Changes from baseline in hs-CRP and IL-6 levels by iron status at baseline and at each yearly time point over the follow-up, defined with soluble transferrin receptor levels when excluding participants who reported kidney disease and liver disease at baseline and participants with incident invasive cancer over the follow-up

|  | **Baseline iron deficiency**  sTfR levels > 28.1 nmol/L | **No baseline iron deficiency**  sTfR levels ≤ 28.1 nmol/L | Mean difference in change from baseline (95% CI) | P value for mean difference in change from baseline between groups ^a^ |
| --- | --- | --- | --- | --- |
|  | n= 535 | n= 1522 |  |  |
| **hs-CRP (mg/L)** |  |  |  |  |
| Unadjusted at baseline, mean (SD) | 3.81 (0.35) | 2.47 (0.11) | 1.33 (0.61 to 2.06) | **<.001** |
| Adjusted change at Year 1 (95% CI) | 0.07 (-0.41 to 0.54) | -0.30 (-0.56 to -0.04) | 0.37 (-0.18 to 0.92) | .19 |
| Adjusted change at Year 2 (95% CI) | 0.15 (-0.37 to 0.67) | 0.00 (-0.36 to 0.38) | 0.14 (-0.50 to 0.78) | .66 |
| Adjusted change at Year 3 (95% CI) | -0.25 (-0.58 to 0.08) | -0.26 (-0.57 to 0.05) | 0.01 (-0.44 to 0.46) | .97 |
| Adjusted change across all time points (95% CI) | -0.01 (-0.31 to 0.29) | -0.19 (-0.39 to 0.02) | 0.17 (-0.19 to 0.54) | .35 |
| **IL-6 (ng/L)** |  |  |  |  |
| Unadjusted at baseline, mean (SD) | 4.56 (0.40) | 3.41 (0.15) | **1.14 (0.30 to 1.99)** | **.008** |
| Adjusted change at Year 1 (95% CI) | **0.92 (0.38 to 1.47)** | **0.35 (0.09 to 0.60)** | 0.58 (-0.05 to 1.20) | .07 |
| Adjusted change at Year 2 (95% CI) | **0.89 (0.38 to 1.40)** | **0.70 (0.40 to 0.99)** | 0.19 (-0.44 to 0.82) | .55 |
| Adjusted change at Year 3 (95% CI) | **1.28 (0.57 to 1.99)** | **0.45 (0.19 to 0.70)** | **0.83 (0.05 to 1.62)** | **.04** |
| Adjusted change across all time points (95% CI) | **1.03 (0.61 to 1.46)** | **0.50 (0.28 to 0.71)** | **0.53 (0.02 to 1.05)** | **.04** |
|  | **Iron deficiency at any yearly follow-up time point**  sTfR levels > 28.1 nmol/L | **No iron deficiency at any yearly follow-up time point**  sTfR levels ≤ 28.1 nmol/L | Mean difference in change from baseline (95% CI) | P value for mean difference in change from baseline between groups ^a^ |
| **hs-CRP (mg/L)** |  |  |  |  |
| Unadjusted at baseline, mean (SD) | 3.89 (0.34) | 2.44 (0.11) | **1.45 (0.75 to 2.15)** | **<.001** |
| Adjusted change at Year 1 (95% CI)^b^ | **0.97 (0.22 to 1.72)** [n= 450] | **-0.58 (-0.81 to -0.35)** [n= 1380] | **1.55 (0.72 to 2.38)** | **<.001** |
| Adjusted change at Year 2 (95% CI) ^b^ | **1.08 (0.37 to 1.78)**  [n= 451] | -0.31 (-0.66 to 0.04) [n= 1311] | **1.39 (0.57 to 2.20)** | **<.001** |
| Adjusted change at Year 3 (95% CI) ^b^ | **1.18 (0.19 to 2.17)** [n=427] | **-0.73 (-0.90 to -0.55)**  [n=1334] | **1.90 (0.85 to 2.96)** | **<.001** |
| Adjusted change across all time points  (95% CI) ^b^ | **1.07 (0.47 to 1.68)** | **-0.54 (-0.71 to -0.36)** | **1.61 (0.94 to 2.29)** | **<.001** |
| **IL-6 (ng/L)** |  |  |  |  |
| Unadjusted at baseline, mean (SD) | 4.52 (0.34) | 3.42 (0.15) | **1.11 (0.42 to 1.79)** | **.002** |
| Adjusted change at Year 1 (95% CI) ^b^ | **1.53 (0.84 to 2.21)** [n=450] | 0.16 (-0.05 to 0.37) [n=1380] | **1.37 (0.64 to 2.10)** | **<.001** |
| Adjusted change at Year 2 (95% CI) ^b^ | **1.47 (0.91 to 2.04)** [n=451] | **0.50 (0.23 to 0.76)** [n=1311] | **0.97 (0.33 to 1.62)** | **.003** |
| Adjusted change at Year 3 (95% CI) ^b^ | **2.00 (1.25 to 2.75)** [n=427] | **0.22 (0.00 to 0.44)** [n=1334] | **1.78 (0.99 to 2.56)** | **<.001** |
| Adjusted change across all time points  (95% CI) ^b^ | **1.67 (1.22 to 2.11)** | **0.29 (0.11 to 0.48)** | **1.37 (0.88 to 1.86)** | **<.001** |

^a^ p-values correspond to the mean differences in biomarkers levels or changes in biomarker levels between iron deficient and non-iron deficient groups. Baseline levels are compared using a t-test. Yearly changes from baseline are compared by repeated measures linear regression with interaction terms between iron deficiency status and time. Overall differences across all time points are compared by repeated measures linear regression with a main effect for iron deficiency.

Models are adjusted for treatment allocation, age, sex, center, body mass index over the follow-up, alcohol consumption, tobacco consumption, polypharmacy, number of comorbidities, frailty status (pre-frailty), frequency of physical activity, yearly incidence rate of infections, baseline level of the outcome and baseline iron status (longitudinal analysis).

Numbers between squared brackets indicate the number of iron-deficient and non-iron deficient participants at each yearly time point

sTfR: soluble Transferrin Receptor

Values in bold indicate significant P-values
